# Development and Validation of Nomograms to Predict Operative Link for Gastritis Assessment Any-Stage and Stages III–IV in the Chinese High-Risk Gastric Cancer Population

**DOI:** 10.3389/fmed.2021.724566

**Published:** 2021-08-10

**Authors:** Song Wang, Fei Ye, Yuan Sheng, Wenyong Yu, Yingling Liu, Dehua Liu, Kaiguang Zhang

**Affiliations:** Department of Gastroenterology, The First Affiliated Hospital of USTC, Division of Life Sciences and Medicine, University of Science and Technology of China, Hefei, China

**Keywords:** gastric atrophy, *Helicobacter pylori*, operative link on gastritis assessment, logistic regression model, nomogram

## Abstract

**Purpose:** It is very essential to diagnose gastric atrophy in the area with high prevalence of gastric cancer. Operative link for gastritis assessment (OLGA) was developed to detect the severity of gastric atrophy. The aim of this study was to develop and validate nomograms for predicting OLGA any-stage and stages III–IV in the Chinese high-risk gastric cancer population.

**Methods:** We retrospectively analyzed 7,945 participants obtained by a multicenter cross-sectional study. We randomly selected 55% individuals (4,370 participants, training cohort) to analyze and generate the prediction models and validated the models on the remaining individuals (3,575 participants, validation cohort). A multivariate logistic regression model was used to select variables in the training cohort. The corresponding nomograms were developed to predict OLGA any-stage and stages III–IV, respectively. The area under the receiver operating characteristic curves and the GiViTI calibration belts were used to estimate the discrimination and calibration of the prediction models.

**Results:** There were 1,226 (28.05%) participants in the training sample and 970 (27.13%) in the validation sample who were diagnosed with gastric atrophy. The nomogram predicting OLGA any-stage had an area under the curve (AUC) of 0.610 for the training sample and 0.615 for the validation sample, with favorable calibrations in the overall population. Similarly, the nomogram predicting OLGA stages III–IV had an AUC of 0.702 and 0.714 for the training and validation samples, respectively, with favorable calibrations in the overall population.

**Conclusions:** The prediction model can early identify the occurrence of gastric atrophy and the severity stage of gastric atrophy to some extent.

## Introduction

Gastric cancer (GC) is a malignant tumor, which is the fifth most common diagnosed cancer and the third cause of death, causes serious disease burden (1). China contributes to the highest incidence and mortality cases of GC (2). Therefore, early detection and prevention are essential for the management of GC. Atrophic gastritis (AG) is the loss of appropriate glands in gastric mucosa, replaced by connective tissue and/or intestinal epithelial cells (3). It is a precancerous disease that predisposes to the development of gastric cancer, and the risk of gastric cancer is raised in patients with advanced AG (4). Rugge et al. proposed the operative link on gastritis assessment (OLGA) system for the classification and stage of gastritis phenotypes, which depends on the histopathological results of endoscopic biopsy samples and provides information on the extent of gastric atrophy (5). The OLGA system has designated high-risk stages III–IV as precancerous lesions which deserves special follow-up (6, 7). Serological tests, such as gastrin 17 (G-17) and pepsinogen (PG), can help identify cases at risk for AG. PG is a protease secreted into gastric cavity and converted into pepsin (8). Two major types of PG, PG I and PG II, can accurately reflect the state of gastric mucosal injury to some extent (9). G-17 is an important gastrointestinal peptide hormone produced by G cells in gastric antrum, which is related to the location and degree of gastric mucosal atrophy (10). *Helicobacter pylori* (Hp), classified as a human carcinogen, is a well-known risk factor for GC (11). It can cause persistent damage to gastric mucosa.

It is recommended to do endoscopic follow-up and gastric biopsy in individuals with AG, even after the eradication of Hp, to detect GC early and reduce mortality (12). However, identifying individuals with potential AG remains a problem. Endoscopy is expensive, uncomfortable, and does not have good patient compliance as screening tests. Some noninvasive tools that can easily identify individuals with AG are essential to improve the early diagnosis of GC. It was reported that PG I, PG II, PG I/II ratio, and G-17 have been used as GC screening tools in East Asia and other areas with high incidence of Hp infection and GC (13).

Our previous study found that there were correlations among PGI, PG I/II ratio (PGR), and OLGA stage, and they can provide important information for the evaluation of gastric atrophy (14). However, there are few studies to investigate the independent factors of OLGA stage. Besides, the risk prediction models of OLGA stage are not clear. Therefore, in this research, we studied the clinical significance of Hp, PG I, PG II, PG I/II ratio, and G-17 values and determined whether these indicators can be used as independent biomarkers in participants with gastric atrophy. In addition, we aimed to establish risk models to predict OLGA any-stage and stages III–IV in Chinese high-risk population of gastric cancer.

## Materials and Methods

### Design and Study Population

**Figure 1:**
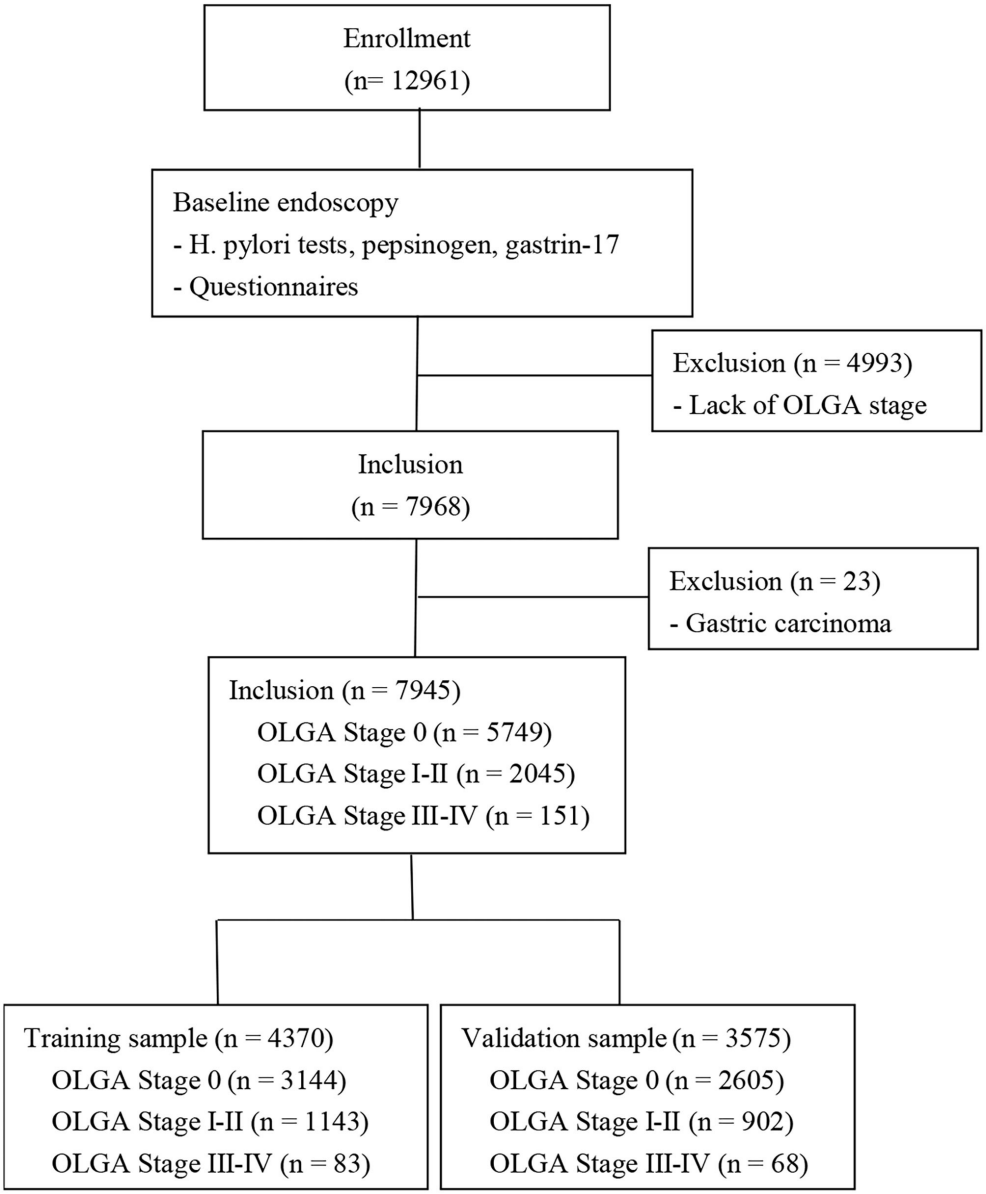
Flow chart for establishing of the training and validation sample. A total of 12,961 participants underwent at our hospital, but 4,993 participants were excluded from the study due to the lack of OLGA stage and 23 participants were excluded due to gastric cancer. Of the remaining 7,945 participants, 4,370 participants were allocated to the training sample and 3,575 participants were allocated to the validation sample. OLGA, operative link for gastritis assessment.

This is a multicenter cross-sectional study, which was approved by the ethics committees of 115 hospitals in China. The inclusion criteria were individuals with “high-risk population of GC” in China. Individuals who met the following criteria were excluded from the study: abdominal mass, unintentional weight loss of more than 4.5 kg over the past 6 months, dysphagia, abdominal pain, emesis, previous Hp eradication, proton pump inhibitors or H2 blocker intake within 2 weeks, pregnancy, cancer history, and endoscopy examination within 1 year. The case-selection flowchart has been summarized in Figure 1.

### Questionnaires

The questionnaire included the following information: age, body mass index, gender, lifestyle [smoking (more than one cigarette a day for more than a year), alcohol drinking (intake of alcohol more than once a week for more than a year)], family history of GC in first-degree relatives, and eating habits (barbecue food, white meat, fresh fruit, green vegetables). White meat includes chicken, duck, and fish. Frequent intake of certain food was defined as eating this kind of food more than three times a week. All participants completed the study survey prior to endoscopy.

### Endoscopy and Histology

Endoscopy was performed by skilled endoscopists in each hospital. Biopsy specimens were collected from body, antrum, angulus, and lesion area of the stomach. At least three biopsy specimens were collected from each participant. The diagnosis was based on the Updated Sydney system (3). All specimens were reviewed by two independent pathologists. Individuals with multiple lesions were classified according to the most severe lesion. Patients who were not diagnosed with gastric atrophy (with or without other diagnosis) during endoscopy were used as controls in this study.

### Serological Testing for Pepsinogen and G-17 and Hp Infection

Serum concentrations of PG I, PG II, G-17, and anti-Hp IgG antibody were tested by commercial ELISA kits (PGI, PGII, G-17, and *Helicobacter pylori* antibody ELISA kits; Biohit, Helsinki, Finland). Carcinoembryonic antigen (CEA) and carbohydrate antigen 199 (CA199) were tested by electrochemiluminescence immunoassay. The detection instrument is Roche cobas 8,000 modular analyzer series (Mannheim, Germany). CEA and CA199 were divided into negative ( ≤ 5 ng/ml) or positive (>5 ng/ml) and negative ( ≤ 37 U/ml) or positive (>37 U/ml), respectively. The test was conducted by the unified training personnel of all qualified laboratories in every hospital. The definition of seropositivity for each indicator was determined by the manufacturer's protocol.

### Cut-Off Values

The original PGs and G-17 concentrations in the nonatrophic gastritis population of the training sample were divided into 20 parts to obtain 20 cut-off values. The prevalence of OLGA stages III–IV for each 20 cut-off values was calculated, and the cut-off value categories with similar prevalence were combined. Two categories of PG I/II ratio and three categories of PG I, PG II, and G-17 were selected. We also adjusted the following covariates: body mass index (BMI) (<18.5, 18.5–23.9, and >23.9 kg/m^2^) and age (40–49, 50–59, 60–69, and >69 years).

### Statistical Analysis

Before the analysis, 55% of the included participants were randomly selected for clinical prediction model (training cohort), and the remaining 45% of the participants were used to verify the model (validation cohort). Categorical variables are reported as counts (percentages) and compared using the Chi-square test. The endpoints of the study were OLGA any-stage and stages III–IV. The risk factors were analyzed by using univariate and multivariate logistic regression analyses. Variables (those with *p* < 0.05 in the univariate analysis) were included in the multivariate logistic regression analysis. The variable was selected by a backward, stepwise method. The final nomogram prediction models were established to calculate the probability of OLGA any-stage and stages III–IV based on the regression coefficients of independent variables. The prediction models were assessed from the perspective of discrimination and calibration. The discrimination of the nomogram was evaluated by calculating the area under the curve (AUC) of the receiver operating characteristic curve (ROC). The calibration of the prediction model was evaluated by GiViTI calibration belt. Statistical analyses were performed using IBM SPSS Statistics for Windows (version 22.0) and R for Windows (version 4.0.4). A twotailed *p*-value <0.05 was considered statistically significant.

## Results

### Individual Demographics

Twelve thousand nine hundred sixty-one patients met the inclusion criteria. However, 4,993 participants were excluded due to the lack of OLGA stage and 23 participants were excluded due to gastric cancer. Of the remaining 7,945 patients, 5,749 (72.36%) had no gastric atrophy and 2,196 (27.64%) had visual gastric atrophy. The training sample included 4,370 randomly selected participants (55%): 3,144 participants without gastric atrophy, 1,143 participants with OLGA stages I–II, and 83 participants with OLGA stages III–IV. The validation sample included 3,575 participants: 2,605 participants without gastric atrophy, 902 participants with OLGA stages I–II, and 68 participants with OLGA stages III–IV. Individual characteristics are summarized in Table 1. There were no differences in the characteristics between the two groups.

**Table 1:** Characteristics of the validation and training cohorts.

	**Training cohort (*N* = 4,370)**	**Validation cohort (*N* = 3,575)**	***p*-value**
**OLGA stage [** ***n*** **(%)]**			
Nonatrophic gastritis	3,144	2,605	0.643
I–II	1,143	902	
III–IV	83	68	
**Age (years)**			
40–49	1,188	993	0.611
50–59	1,499	1,199	
60–69	1,215	1,023	
>69	468	360	
**Body mass index**			
≤ 18.5	240	208	0.225
18.5–23.9	2,632	2,206	
>23.9	1,498	1,161	
**PG I (ng/ml)**			
≤ 46.13	261	218	0.704
46.13–103.49	1,759	1,406	
>103.49	2,350	1,951	
**PG II (ng/ml)**			
≤ 2.88	203	167	0.783
2.88–18.2	3,274	2,655	
>18.2	893	753	
**PG I/II ratio**			
≥5.75	3,868	3,136	0.277
<5.75	502	439	
**G-17 (pmol/L)**			
≤ 3.93	2,349	1,881	0.371
3.93–17.31	1,335	1,093	
>17.31	686	601	
**Sex**			
Female	2,233	1,839	0.761
Male	2,137	1,736	
***Helicobacter pylori***			
Negative	2,521	2,001	0.124
Positive	1,849	1,574	
**CEA**			
Negative	4,223	3,442	0.391
Positive	147	133	
**CA199**			
Negative	4,217	3,441	0.557
Positive	153	134	
**Family history**			
No	3,800	3,103	0.834
Yes	570	472	
**Diabetes**			
No	4,156	3,407	0.682
Yes	214	168	
**Hypertension**			
No	3,720	3,041	0.938
Yes	650	534	
**Smoking**			
No	3,375	2,744	0.616
Yes	995	831	
**Alcohol drinking**			
No	3,595	2,935	0.846
Yes	775	640	
**Barbecue food**			
Occasional	4,207	3,450	0.580
Regular	163	125	
**White meat**			
Occasional	2,441	1,959	0.344
Regular	1,929	1,616	
**Green vegetables**			
Occasional	857	687	0.659
Regular	3,513	2,888	
**Fresh fruits**			
Occasional	2,074	1,673	0.556
Regular	2,296	1,902	

*Values are presented as n (%). For the variables of eating habits, two types of consumption frequency were provided, namely, occasional (<3 times a week) and regular (at least three times a week). OLGA, operative link for gastritis assessment; CEA, carcinoembryonic antigen; CA199, carbohydrate antigen199; PG, pepsinogen; G-17, gastrin-17*.

### Nomogram Development

The characteristics of participants with nonatrophic gastritis OLGA stages I–II and stages III–IV in training samples are summarized in Table 2. There were significant differences in age (*p* < 0.001), BMI (*p* = 0.038), PG I (*p* < 0.001), PG II (*p* = 0.005), PG I/II ratio (*p* < 0.001), G-17 (*p* = 0.020), Hp infection (*p* = 0.001), CEA (*p* = 0.017), CA199 (*p* = 0.031), white meat (*p* < 0.001), and fresh fruits (*p* = 0.042) among the three subgroups.

**Table 2:** Characteristics and diagnostic test results associated with nonatrophic gastritis, OLGA stages I–II and III–IV in the training sample.

	**Nonatrophic gastritis**	**Stages I–II**	**Stages III–IV**	***p*-value**
**Age (years)**				
40–49	932	239	17	<0.001
50–59	1,087	387	25	
60–69	832	356	27	
>69	293	161	14	
**Body mass index**				
≤ 18.5	160	71	9	0.038
18.5–23.9	1,876	708	48	
>23.9	1,108	364	26	
**PG I (ng/ml)**				
≤ 46.13	158	89	14	<0.001
46.13–103.49	1,257	474	28	
>103.49	1,729	580	41	
**PG II (ng/ml)**				
≤ 2.88	158	39	6	0.005
2.88–18.2	2,358	866	50	
>18.2	628	238	27	
**PG I/II ratio**				
≥5.75	2,829	976	63	<0.001
<5.75	315	167	20	
**G-17 (pmol/L)**				
≤ 3.93	1,730	582	37	0.020
3.93–17.31	943	358	34	
>17.31	471	203	12	
**Sex**				
Female	1,621	575	37	0.374
Male	1,523	568	46	
***Helicobacter pylori***				
Negative	1,846	643	32	0.001
Positive	1,298	500	51	
**CEA**				
Negative	3,053	1,092	78	0.017
Positive	91	51	5	
**CA199**				
Negative	3,032	1,109	76	0.031
Positive	112	34	7	
**Family history**				
No	2,734	995	71	0.925
Yes	410	148	12	
**Diabetes**				
No	2,989	1,089	78	0.858
Yes	155	54	5	
**Hypertension**				
No	2,671	981	68	0.553
Yes	473	162	15	
**Smoking**				
No	2,431	888	56	0.098
Yes	713	255	27	
**Alcohol drinking**				
No	2,579	946	70	0.756
Yes	565	197	13	
**Barbecue food**				
Occasional	3,016	1,109	82	0.116
Regular	128	34	1	
**White meat**				
Occasional	1,661	727	53	<0.001
Regular	1,483	416	30	
**Green vegetables**				
Occasional	597	242	18	0.251
Regular	2,547	901	65	
**Fresh fruits**				
Occasional	1,456	579	39	0.042
Regular	1,688	564	44	

*Values are presented as n (%). For the variables of eating habits, two types of consumption frequency were provided, namely, occasional (<3 times a week) and regular (at least three times a week). OLGA, operative link for gastritis assessment; CEA, carcinoembryonic antigen; CA199, carbohydrate antigen199; PG, pepsinogen; G-17, gastrin-17*.

**Figure 2:**
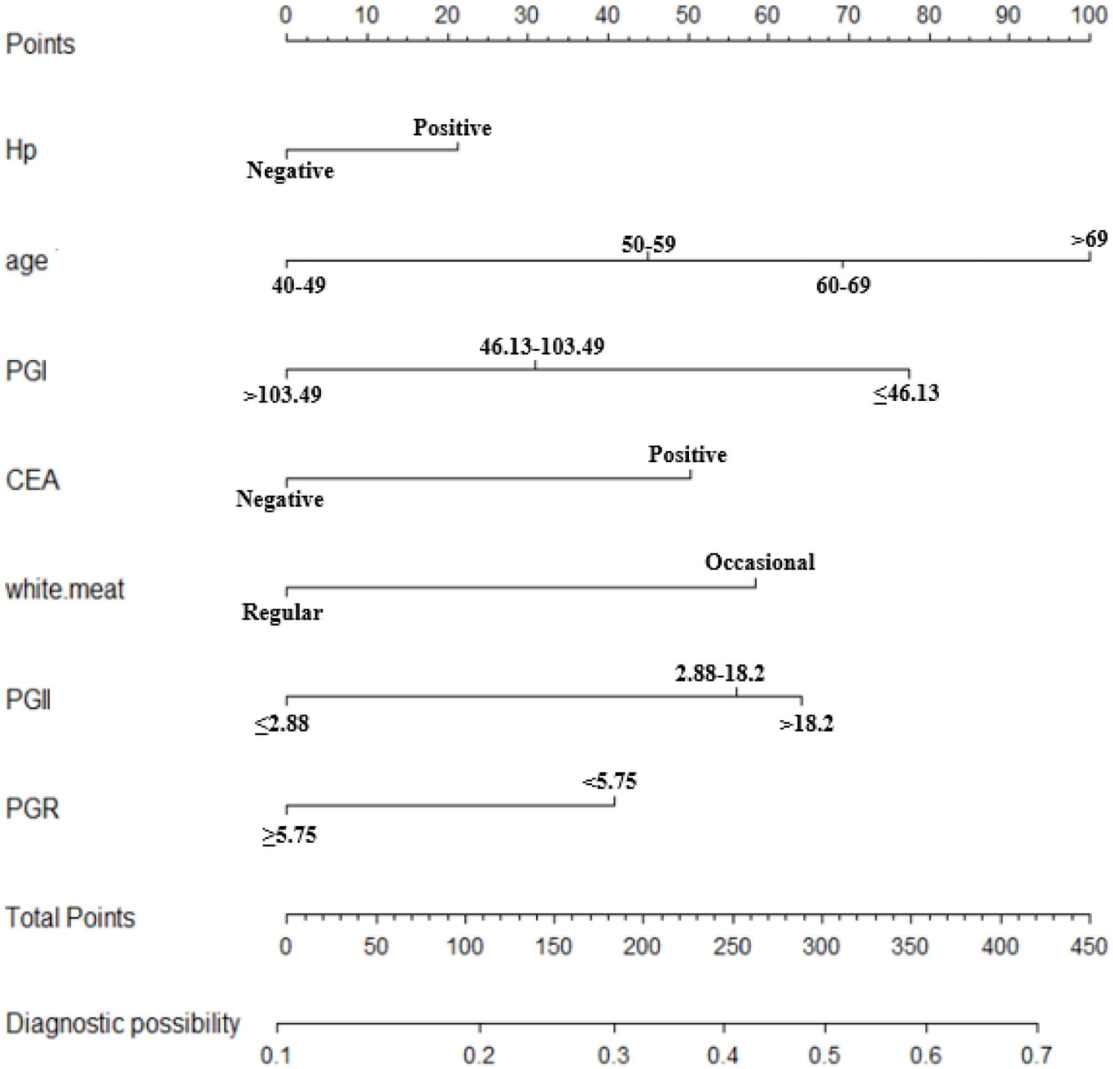
Nomogram to predict the probability of OLGA any-stage in the Chinese high-risk gastric cancer population. *For the variables of eating habits (white.meat), two types of consumption frequency were provided, namely, occasional (<3 times a week) and regular (at least three times a week). OLGA, operative link for gastritis assessment; Hp, *Helicobacter pylori*; CEA, carcinoembryonic antigen; PG, pepsinogen; PGR, PG I/II ratio.

In order to determine the independent factors of the models, we conducted a logistic regression analysis with the backward, stepwise selection of variables. Variables with a *p*-value <0.05 in univariate analyses entered into the multivariate models (Table 3). Age, PG I, PG II, PG I/II ratio, CEA, Hp infection, and white meat were independent risk factors of participants with OLGA any-stage (*p* < 0.05). In the model of OLGA stages III–IV, six variables were selected: age, PG I, PGR, smoking, Hp infection, and white meat. The nomographs of the two models are shown in Figures 2, 4, respectively. On the basis of nomograms, we can obtain the corresponding points of each predictive index, and the sum of the points is calculated as the total score. The predictive risk corresponding to the total score is the probability of OLGA any-stage and stages III–IV.

**Table 3:** Logistic regression model showing the association of variables with OLGA any-stage and OLGA stages III–IV.

	**OLGA any-stage**				**OLGA stages III–IV**			
	**Univariable**		**Multivariable**		**Univariable**		**Multivariable**	
	**OR (95% CI)**	***p*-value**	**OR (95% CI)**	***p-*value**	**OR (95% CI)**	***p*-value**	**OR (95% CI)**	***p*-value**
Age (years)	–	–	–	–	–	–	–	–
40–49	–	–	–	–	–	–	–	–
50–59	1.380 (1.154–1.650)	<0.001	1.379 (1.151–1.653)	<0.001	1.261 (0.677–2.349)	0.465	1.222 (0.652–2.289)	0.532
60–69	1.676 (1.395–2.014)	<0.001	1.640 (1.361–1.976)	<0.001	1.779 (0.963–3.287)	0.066	1.742 (0.935–3.244)	0.080
>69	2.174 (1.723–2.744)	<0.001	2.044 (1.612–2.592)	<0.001	2.620 (1.276–5.379)	0.009	2.423 (1.160–5.058)	0.018
Body mass index	–	–	–	–	–	–	–	–
≤ 18.5	–	–	–	–	–	–		
18.5–23.9	0.806 (0.608–1.068)	0.133	–	–	0.455 (0.219–0.944)	0.034	–	–
>23.9	0.704 (0.526–0.943)	0.019	–	–	0.417 (0.192–0.906)	0.027		
PG I (ng/ml)	–	–	–	–	–	–	–	–
≤ 46.13	–	–	–	–	–	–	–	–
46.13–103.49	0.613 (0.468–0.802)	<0.001	0.717 (0.530–0.971)	0.031	0.251 (0.130–0.488)	<0.001	0.362 (0.170–0.771)	0.008
>103.49	0.551 (0.423–0.718)	<0.001	0.575 (0.419–0.789)	0.001	0.268 (0.143–0.502)	<0.001	0.311 (0.152–0.639)	0.001
PG II (ng/ml)	–	–	–	–	–	–	–	–
≤ 2.88	–	–	–	–	–	–	–	–
2.88–18.2	1.364 (0.971–1.916)	0.073	1.492 (1.045–2.131)	0.028	0.558 (0.236–1.322)	0.185	–	–
>18.2	1.482 (1.033–2.126)	0.033	1.582 (1.048–2.389)	0.029	1.132 (0.460–2.789)	0.787	–	–
PG I/II ratio	–	–	–	–	–	–	–	–
≥5.75	–	–	–	–	–	–	–	–
<5.75	1.616 (1.331–1.963)	<0.001	1.339 (1.053–1.704)	0.017	2.851 (1.701–4.778)	<0.001	1.741 (0.959–3.160)	0.068
G-17 (pmol/L)	–	–	–	–	–	–	–	–
≤ 3.93	–	–	–	–	–	–	–	–
3.93–17.31	1.162 (1.001–1.349)	0.049	–	–	1.686 (1.051–2.704)	0.030	–	–
>17.31	1.276 (1.060–1.536)	0.010	–	–	1.191 (0.616–2.303)	0.603	–	–
Sex	–	–	–	–	–	–	–	–
Female	–	–	–	–	–	–	–	–
Male	1.068 (0.936–1.219)	0.330	–	–	1.323 (0.854–2.051)	0.211	–	–
*Helicobacter pylori*	–	–	–	–	–	–	–	–
Negative	–	–	–	–	–	–	–	–
Positive	1.161 (1.016–1.326)	0.028	1.165 (1.015–1.337)	0.030	2.267 (1.449–3.546)	<0.001	2.363 (1.491–3.743)	<0.001
CEA	–	–	–	–	–	–	–	–
Negative	–	–	–	–	–	–	–	–
Positive	1.606 (1.143–2.255)	0.006	1.433 (1.012–2.028)	0.042	2.151 (0.850–5.439)	0.106	–	–
CA199	–	–	–	–	–	–	–	–
Negative	–	–	–	–	–	–	–	–
Positive	0.937 (0.651–1.348)	0.725			2.493 (1.124–5.531)	0.025	–	–
Family history	–	–	–	–	–	–	–	–
No	–	–	–	–	–	–	–	–
Yes	1.001 (0.823–1.217)	0.993	–	–	1.127 (0.606–2.096)	0.706	–	–
Diabetes	–	–	–	–	–	–	–	–
No	–	–	–	–	–	–	–	–
Yes	0.975 (0.717–1.326)	0.871	–	–	1.236 (0.493–3.097)	0.651	–	–
Hypertension	–	–	–	–	–	–	–	–
No	–	–	–	–	–	–	–	–
Yes	0.953 (0.790–1.149)	0.612	–	–	1.246 (0.706–2.197)	0.448	–	–
Smoking	–	–	–	–	–	–	–	–
No	–	–	–	–	–	–	–	–
Yes	1.019 (0.871–1.192)	0.819	–	–	1.644 (1.031–2.622)	0.037	1.712 (1.061–2.762)	0.028
Alcohol drinking	–	–	–	–	–	–	–	–
No	–	–	–	–	–	–	–	–
Yes	0.943 (0.793–1.123)	0.513	–	–	0.848 (0.466–1.543)	0.589	–	–
Barbecue food	–	–	–	–	–	–	–	–
Occasional	–	–	–	–	–	–	–	–
Regular	0.692 (0.474–1.012)	0.058	–	–	0.287 (0.040–2.081)	0.217	–	–
White meat	–	–	–	–	–	–	–	–
Occasional	–	–	–	–	–	–	–	–
Regular	0.640 (0.559–0.734)	<0.001	0.658 (0.574–0.756)	<0.001	0.634 (0.403–0.998)	0.049	0.665 (0.420–1.054)	0.083
Green vegetables	–	–	–	–	–	–	–	–
Occasional	–	–	–	–	–	–	–	–
Regular	0.871 (0.740–1.025)	0.097	–	–	0.846 (0.498–1.437)	0.537	–	–
Fresh fruits	–	–	–	–	–	–	–	–
Occasional	–	–	–	–	–	–	–	–
Regular	0.849 (0.744–0.968)	0.015	–	–	0.973 (0.629–1.506)	0.903	–	–

*Values are presented as n (%). For the variables of eating habits, two types of consumption frequency were provided, namely, occasional (<3 times a week) and regular (at least three times a week). OLGA, operative link for gastritis assessment; CEA, carcinoembryonic antigen; CA199, carbohydrate antigen199; PG, pepsinogen; G-17, gastrin-17*.

### Nomogram Validation

**Figure 3:**
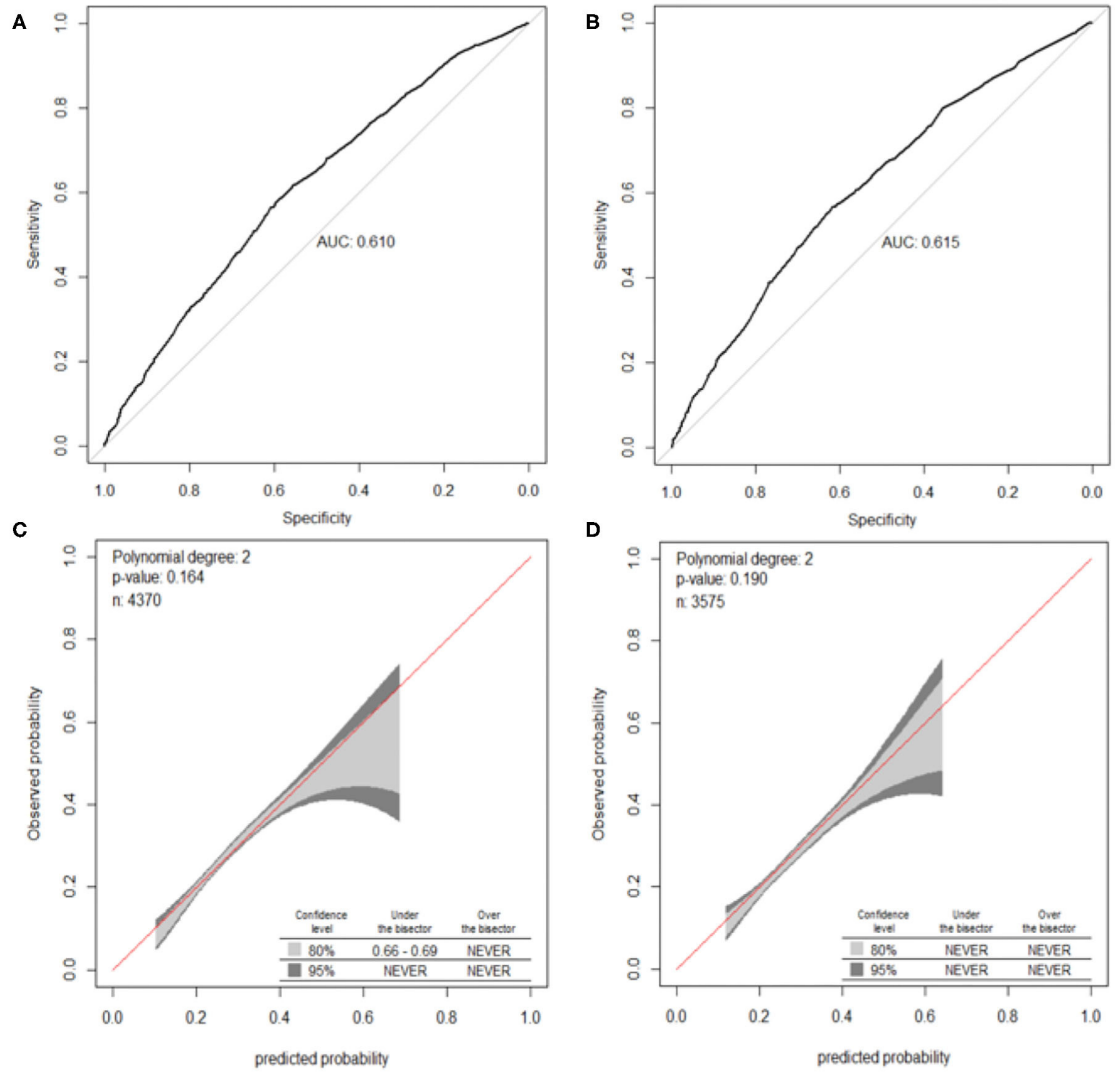
ROC curves and calibration plots of the nomogram for OLGA any-stage. **(A)** ROC curve in the training cohort, **(B)** ROC curve in the validation cohort, **(C)** calibration plot in the training cohort, and **(D)** calibration plot in the validation cohort. OLGA, operative link for gastritis assessment; ROC, receiver operating characteristic curve; AUC, area under the curve.

The validation of the nomograms was based on discrimination and calibration. The AUCs of the nomogram for OLGA any-stage were 0.610 [95% confidence interval (CI), 0.592–0.628] in the training sample and 0.615 (95% CI, 0.595–0.636) in the validation sample. The 95% of CIs of GiViTi calibration belt in the training sample and the validation sample did not pass through the diagonal bisector line and the *p*-values of the two groups of GiVITI calibration test were 0.164 and 0.190, respectively (Figure 3). Therefore, the prediction probability of the model is consistent with the actual probability, which shows that the prediction model has strong consistency, and the calibration results of the two groups of prediction models are favorable. The AUCs of the nomogram for OLGA stages III–IV were 0.702 (95% CI, 0.646–0.758) in the training sample and 0.714 (95% CI, 0.647–0.781) in the validation sample. The 95% of CIs of GiViTi calibration belt in the training sample and the validation sample did not pass through the diagonal bisector line. The *p*-values of the two groups of GiVITI calibration test were 0.892 and 0.753, respectively (Figure 5). The model displayed good calibration in the OLGA III–IV population.

**Figure 4:**
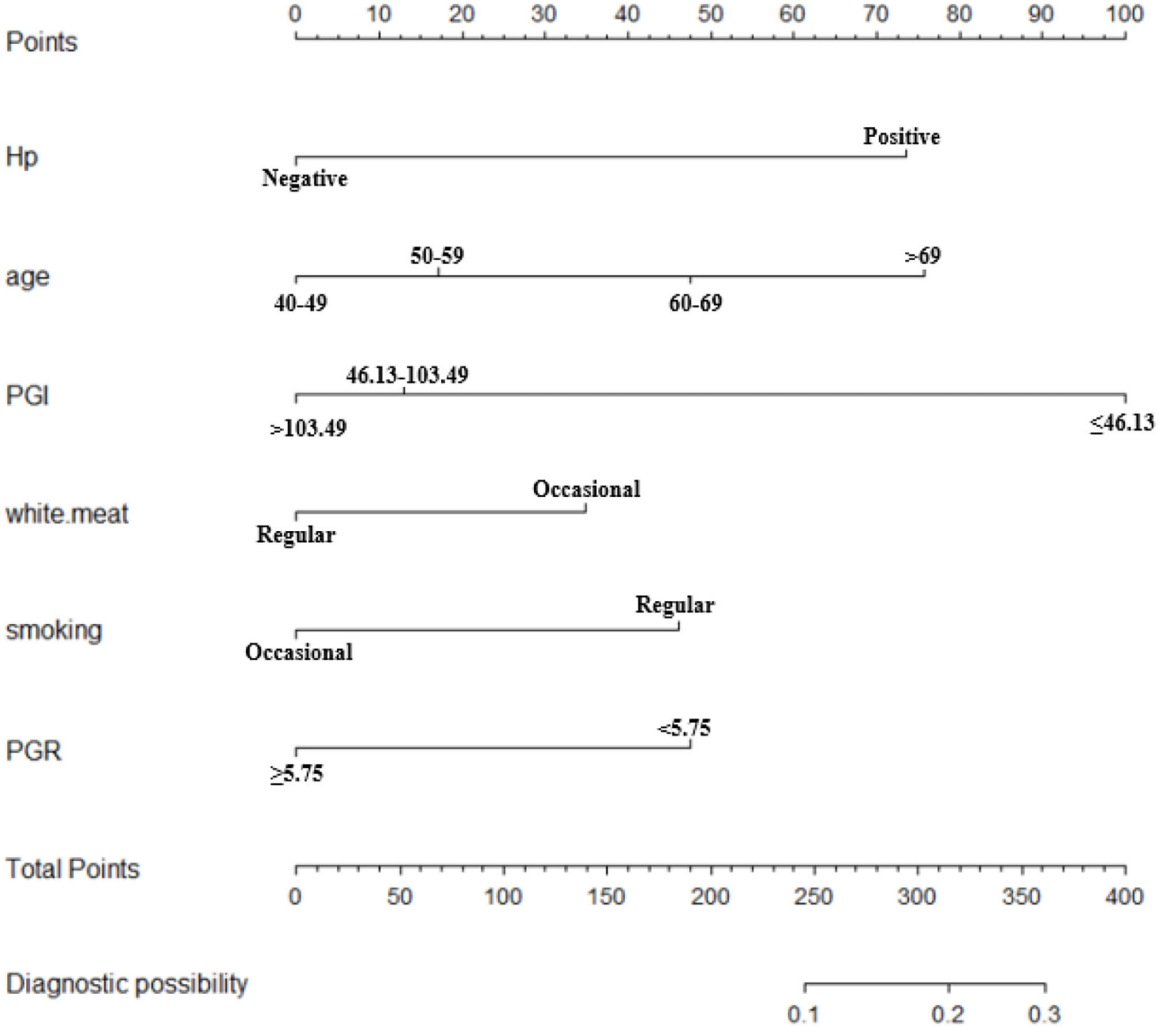
Nomogram to predict the probability of OLGA stages III–IV in the Chinese high-risk gastric cancer population. *For the variables of eating habits (white.meat), two types of consumption frequency were provided, namely, occasional (<3 times a week) and regular (at least three times a week). OLGA, operative link for gastritis assessment; Hp, *Helicobacter pylori*; PG, pepsinogen; PGR, PG I/II ratio.

**Figure 5:**
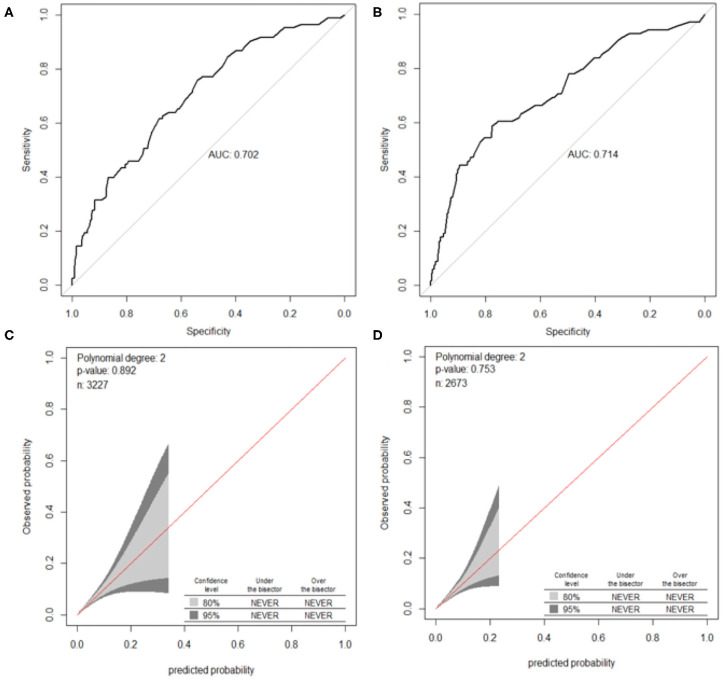
ROC curves and calibration plots of the nomogram for OLGA stages III–IV. **(A)** ROC curve in the training cohort, **(B)** ROC curve in the validation cohort, **(C)** calibration plot in the training cohort, and **(D)** calibration plot in the validation cohort. OLGA, operative link for gastritis assessment; ROC, receiver operating characteristic curve; AUC, area under the curve.

## Discussion

The incidence and mortality rate of GC have fallen, and survival rates have generally improved over the past several decades (2). However, nearly half of GC participants occurred in China and the prognosis remains poor (2, 15). The progressive changes of the gastric mucosa result in intestinal type GC and the gastric mucosal atrophy is associated with increased cancer risk (4, 16, 17). Early identification of gastric precancerous lesion, such as AG, may improve gastric cancer detection and prevention. AG is generally asymptomatic. In our study, the occurrence rate of gastric atrophy was 27.6% and the rate of severe atrophy was 1.9%. The OLGA staging system is used to classify and grade the severity and distribution of atrophy. Severe atrophy (OLGA stages III–IV) is associated with an increased risk of gastric neoplasm (5, 18, 19). Gastroscopy, a costly invasive procedure, is difficult to perform. Thus, for the assessment of GC risk, the opportunistic screening is a kind of clinical screening, which could expand the coverage of the screening and increase the early diagnosis rate of GC, to improve the 5-year survival rate of GC patients and achieve the sustainable development of the cancer prevention and treatment. In this study, we attempted to develop and validate noninvasive prediction models to evaluate the morphologic and functional status of the gastric mucosa in patients with AG. Thus, we developed nomograms to predict OLGA any-stage and stages III–IV in the Chinese high-risk gastric cancer population and validated the performance of nomograms in all subjects.

Hp infection has been recognized as the main environmental promoter of carcinogenic process (11, 12). Hp has a significant correlation with AG, intestinal metaplasia, dysplasia, mucosa-associated lymphoid tissue (MALT) lymphoma, and GC. More than 50% of the world population was infected with Hp and <2% developed GC, and Hp was responsible for ~90% of the GC participants (11, 20). Compared with patients with a low-negative anti-Hp IgG titer ( ≤ 3 U/ml), patients with a high-negative titer of anti-Hp IgG (3– <10 U/ml) showed a significantly elevated risk of GC in Japanese subjects (21). After 2,000, the infection rate of Hp was lower than before in Europe, Northern America, and Oceania, but the rate was similar in Asia, Latin America, and the Caribbean (22). In the present study, the prevalence of Hp was 43.0% in China, which was similar with previous data (41.35–72.3% according to differences in the study population and geographic region) (23). There were significant differences in Hp prevalence among the nonatrophic gastritis, OLGA stages I–II and stages III–IV subgroups. We also conducted a logistic regression analysis with the backward, stepwise selection of variables. Hp infection was one of the independent risk factors of participants with OLGA any-stage and OLGA stages III–IV.

The tumor markers CEA and CA19-9 levels could provide useful information for the diagnosis of GC patients. In our study, logistic regression analysis showed that CEA levels were significantly related to OLGA any-stage. Gastric mucosal atrophy results in the decrease of serum PGI and PGR levels, and serum pepsinogen tests have been proposed to assess the risk of gastric atrophy and to stratify the risk of GC development (24, 25). The serum PGI and PGR levels decreased significantly with the progression of histological AG, and serum PGI/II ratio decreased significantly with the severity of atrophy (26). The serum PGR are correlated inversely with OLGA stage (18). Hp infection could cause an elevation of PGI, PGII, and G-17 levels, while the PGR was markedly decreased (14, 27). In the asymptomatic physical examination population, serum PGI, PGII, and PGR levels decreased with increasing OLGA scores (28). G-17 is a predominant form of antral hormone gastrins in plasma or tissue in antral mucosa, which can regulate gastric acid secretion and gastric mucosa growth. Wang et al. reported that elevated serum G-17 level was significantly associated with an increased risk of AG in healthy people (29). However, the diagnostic ability of serum G-17 level for AG decreased at higher levels during the whole process of gastric disease (29). In the present study, there were significant differences in PG I, PG II, PGR, and G-17 among the nonatrophic gastritis, OLGA stages I–II and III–IV subgroups. Logistic regression analysis showed that PG I, PG II, and PGR were significantly related to OLGA any-stage. In the model of OLGA stages III–IV, PG I and PGR were independent risk factors.

Dietary habits have been considered an essential risk factor for GC (30). Meta-analysis demonstrated white meat consumption was significantly negatively correlated with the risk of GC (31). In this study, the intake of white meat was independent and a significant protective factor for OLGA any-stage (OR = 0.658; 95% CI, 0.574–0.756). Less heme iron and a rich source of polyunsaturated fatty acids (PUFAs) in white meat may hinder the increased risk of gastric atrophy. PUFAs inhibit the synthesis of interleukin-1 and tumor necrosis factor and reduce the risk of GC (32). A recent study showed that the incidence rate and mortality rate of GC in China were concentrated in the elderly population (especially those aged 60 or above) (33). It was consistent with our results. We also found that BMI of subjects with OLGA any-stage and stages III–IV was significantly lower than that of subjects without gastric atrophy. However, BMI was not an independent factor. Few studies have investigated the association between atrophic BMI and gastritis. One research suggested that the lower BMI observed in patients with extensive atrophic gastritis may be associated with lower serum ghrelin levels (34). It is generally known that people are at high risk of gastric atrophy if they have the family history. However, there were no relationships between them in our study. It is worth a further study. Smoking was another potential indicator of OLGA stages III–IV. It was known that smoking was related to the development of GC precancerous lesions (33). Tobacco smoke contains a variety of chemical carcinogens, which may affect the normal function of DNA by binding to it and eventually lead to GC (35).

The intuitive, easy-to-use, and high-accuracy features of nomogram contribute to its wide application and improve its efficacy in the diagnosis of OLGA any-stage and stages III–IV. The models of OLGA any-stage and stages III–IV showed that there were differences in the predictors and performance in different populations. There were seven variables in the model of OLGA any-stage and six variables were included in OLGA stages III–IV model (Table 3). The AUCs of the two models showed an ability to separate individuals who were with nonatrophic gastritis and OLGA any-stage and with nonatrophic gastritis and OLGA stages III–IV. We predicted that the model of OLGA any-stage had a modest discriminatory ability, and the AUC of the training sample and validation sample was 0.610 and 0.615, respectively. The model developed to predict OLGA stages III–IV also had good differentiation. The AUC of the training cohort was 0.702, and the AUC of the validation cohort was 0.714. In this study, the GiViTI calibration belts showed deviations depending on the population and the severity of gastric atrophy. The model of OLGA any-stage and OLGA stages III–IV model showed good calibrations. There were no significant differences between the prediction probabilities and the observation probabilities.

Some limitations exist in this study. First, since this was a multicenter cross-sectional study, there may have been some bias since people with missing data were automatically excluded from the analysis. Second, external validation of our prediction model is needed. We will apply the same methodology prospectively to evaluate the real clinical efficacy of the nomogram in the further study. Third, the model of OLGA any-stage shows a modest discriminatory ability, which suggests that the role of the model is limited. We attribute the discriminatory ability of the OLGA any-stage model to two factors. First, predicting gastric atrophy is a challenge, especially when compared with other predictive tasks such as OLGA stages III–IV. As mentioned before, some predictive factors (smoking, dietary habits, and tumor markers) are closely related to the occurrence of GC. However, only high-risk OLGA stages III–IV is considered precancerous lesions. Thus, the predictive ability of these known risk factors in the OLGA any-stage model is limited. Second, our study was a multicenter study, which confirmed that, although more complex methods had potential, when the data contained relatively few predictive factors and the sample size is moderate, they did not necessarily bring advantages to the OLGA any-stage model.

## Conclusion

In conclusion, we developed two nomogram models composed of personal characteristics, laboratory parameters, and dietary habits to predict OLGA any-stage and OLGA stages III–IV in Chinese high-risk GC population. The prediction models contribute to improve the early identification and screening of such population. Besides, the models may reduce the need for endoscopy examination and we can predict the presence and severity of gastric atrophy through them.

## Data Availability Statement

The raw data supporting the conclusions of this article will be made available by the authors, without undue reservation.

## Ethics Statement

The studies involving human participants were reviewed and approved by the First Affiliated Hospital of USTC. The patients/participants provided their written informed consent to participate in this study.

## Author Contributions

SW and KZ conceived and designed the study and wrote the manuscript. FY, YS, WY, YL, and DL collected the data. SW, FY, and KZ read, corrected, and approved the final manuscript. All authors contributed to the article and approved the submitted version.

## Conflict of Interest

The authors declare that the research was conducted in the absence of any commercial or financial relationships that could be construed as a potential conflict of interest.

## Publisher's Note

All claims expressed in this article are solely those of the authors and do not necessarily represent those of their affiliated organizations, or those of the publisher, the editors and the reviewers. Any product that may be evaluated in this article, or claim that may be made by its manufacturer, is not guaranteed or endorsed by the publisher.
